# Intrasexual aggression reduces mating success in field crickets

**DOI:** 10.1002/ece3.10557

**Published:** 2023-10-01

**Authors:** Eleanor K. Tinsley, Nathan W. Bailey

**Affiliations:** ^1^ School of Biology University of St Andrews St Andrews UK; ^2^ Institute of Zoology Zoological Society London London UK; ^3^ University College London London UK

**Keywords:** acceptance threshold, aggression, aggressive spillover, behavioural repeatability, mate choice plasticity, repeatability

## Abstract

Aggressive behaviour is thought to have significant consequences for fitness, sexual selection and the evolution of social interactions, but studies measuring its expression across successive encounters—both intra‐ and intersexual—are limited. We used the field cricket *Teleogryllus oceanicus* to evaluate factors affecting repeatability of male aggression and its association with mating success. We quantified focal male aggression expressed towards partners and received from partners in three successive, paired trials, each involving a different male partner. We then measured a proxy of focal male fitness in mating trials with females. The likelihood and extent of aggressive behaviour varied across trials, but repeatability was negligible, and we found no evidence that patterns of focal aggression resulted from interacting partner identity or prior experience. Males who consistently experienced aggression in previous trials showed decreased male mating ‘efficiency’—determined by the number of females a male encountered before successfully mating, but the effect was weak and we found no other evidence that intrasexual aggression was associated with later mating success. During mating trials, however, we observed unexpected male aggression towards females, and this was associated with markedly decreased male mating efficiency and success. Our findings suggest that nonadaptive aggressive spillover in intersexual mating contexts could be an important but underappreciated factor influencing the evolution of intrasexual aggression.

## Graphical abstract

We used the field cricket *Teleogryllus oceanicus* to evaluate factors affecting expression of intrasexual male aggression and mating success; but repeatability, partner and prior experience effects were negligible. By contrast, male aggression towards females in mating trials was associated with markedly decreased male mating success, consistent with nonadaptive aggressive spillover in intersexual mating contexts.

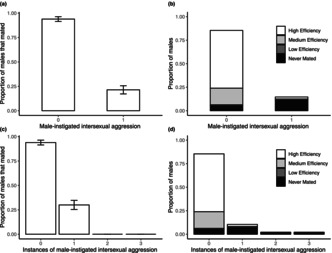

## 1 INTRODUCTION

Intraspecific aggression is virtually ubiquitous across animal taxa (Holekamp & Strauss, 2016), yet there is mixed evidence about the exact nature of its fitness benefits (Ariyomo & Watt, 2012; Cain & Langmore, 2016; Gilby et al., 2013; King et al., 2019; Krippel et al., 2017; McDonald et al., 2017). Although there has been considerable investigation of factors that influence aggression, such as behavioural syndromes (Santostefano et al., 2016; Thys et al., 2017), resource quality (James & Furukawa, 2020) and population density (Knell, 2009), such work tends not to observe whether and how consistently individuals express aggression during successive encounters with different interacting partners and what the consequences are for subsequent mating success.

Repeatability and flexibility of aggression can be thought of as a continuum, rather than mutually exclusive properties of behaviour. In cases where adaptive benefits of aggression are highly context‐dependent, the expression of aggression could be subject to strong influences of interacting partners. In this case, flexibility of aggression within focal individuals might be favourable, whereas the tendency for interacting partners to induce expression of aggression in others might show higher repeatability. To evaluate such scenarios, it is necessary to test whether fitness covaries with both the prior expression of aggression, and the experience of aggression instigated by past and present interacting partners. There is evidence that the type and intensity of intrasexual aggression expressed by an individual can be modified by their prior experience. For example, Yang et al. (2001) suggested that an interaction between habituation and reinforcement causes individuals to exhibit aggression towards unfamiliar conspecifics, and Hsu et al. (2006) emphasised the importance of integrating multiple experiences into aggressive encounter outcome prediction models. Thus, individuals' past experience of aggressive bouts with conspecifics might not only influence their fighting ability, but also whether or not they later engage in aggressive behaviour at all and for how long they invest in those subsequent aggressive bouts. Winner/loser effects in insects, for example, have been commonly cited as predictors of mating success due to their impact on male courtship (Thomas & Simmons, 2008), although not sperm quality (Tuni et al., 2019). Such experiences may also influence the expression of future aggressive behaviours (Abe et al., 2021; Rillich & Stevenson, 2011).

Flexibility of aggression itself could be context‐dependent, such that an individual might show consistent aggressive responses across environmental or social contexts, or only within certain contexts (Bell, 2006; Bengston et al., 2018; Betini & Norris, 2012). In addition, behaviour that is adaptive in one context may ‘spillover’, that is be expressed in, another context in which it is not, affecting its total fitness consequences. A prominent example is aggressive spillover (Arnqvist & Henriksson, 1997; Duckworth, 2006; Johnson & Sih, 2005; Wijnhorst, 2017). The aggressive spillover hypothesis was developed in the context of sexual cannibalism, wherein a particularly aggressive genotype, or ‘personality’, may be selectively advantageous during foraging but disadvantageous during mating (Arnqvist & Henriksson, 1997). In this scenario, a lack of behavioural flexibility due to genetic or environmental constraint may provide the selective context in which sexual cannibalism evolves; evidence for vs. against this hypothesis is subject to debate (Kralj‐Fišer et al., 2012, 2013). Aggressive spillover has been less frequently studied outside the context of sexual cannibalism but may have similarly important consequences for understanding the fitness consequences and evolution of aggression.

Here, we use a laboratory insect model with a well‐characterised repertoire of aggressive behaviours—field crickets—to investigate factors predicting males' tendency to express aggression. Using the field cricket *Teleogryllus oceanicus*, we examined its intra‐individual repeatability (Scherer et al., 2018), estimating variation due to focal versus partner effects and the role of prior experience of aggression. Aggression is common in field crickets of the subfamily Gryllinae. It takes the form of several distinct behaviours that have been almost exclusively studied in male–male encounters: aggressive calling, antennal interaction, charging, mandibular contact and sparring (Alexander, 1961; Bailey & French, 2012; Bertram et al., 2011; Bunting & Hedrick, 2018; Fisher et al., 2019; Fuentes & Shaw, 1986; Hedrick & Bunting, 2014; Logue et al., 2010). Given that it is known to be adaptive in some contexts but thought to incur large fitness costs (Judge & Bonanno, 2008; Kuriwada, 2017), we were particularly interested in how previous patterns of intrasexual aggression related to male mating success.

As aggression is an interacting phenotype whose expression could be expected to depend on properties of interacting partners (cf. Moore et al., 1997; Wilson et al., 2009), we tested the prediction that male aggression should be context‐dependent: We expected that previous aggressive encounters should alter the likelihood of future aggressive encounters and that partner effects should be detectable. We expected this would be reflected by low intra‐individual repeatability across these contexts. The second prediction was that males who were more aggressive towards other males would have higher fitness in subsequent intersexual encounters because greater aggression indicates better fighting ability and thus condition, resource acquisition or genetic quality, translating to higher reproductive success on average. However, an alternative is possible in which greater aggression depletes male resources in a manner that negatively impacts mating success. Another alternative is that males that can express flexible aggression adaptively to optimise the outcome of social encounters should have higher mating success. To distinguish among these, we measured using the proxies of both mating success (i.e. the male did or did not mate) and mating efficiency (i.e. how readily he mated when presented with females—the inverse of the number of females he encountered before mating, to a limit of 3). We did not find support for any of these predictions, though experiencing aggression was weakly associated with decreased mating success. However, we observed unexpected but persistent intersexual aggression instigated by males. Intersexual aggression was associated with decreased male mating success, consistent with aggressive spillover. Our results highlight the importance of considering the total fitness effects of aggression across contexts, including those in which it has rarely been studied. Aggressive spillover during mating may significantly constrain the evolution of male–male aggression in this and other species.

## 2 MATERIALS AND METHODS

### 2.1 Cricket origins and rearing

Experimental crickets were sourced from a large (ca. 100 reproducing individuals), freely breeding laboratory stock population of *T. oceanicus* originating from Townsville, Australia, in March 2013. Stock were maintained in transparent plastic 14‐L containers (40 × 29 × 20 cm), at 25°C on a photo‐reversed 12‐h day–night cycle. Maintenance was performed twice weekly with ad libitum water, food (Burgess Excel Junior and Dwarf rabbit pellets) and egg cartons for shelter replaced. Due to the well‐established impact of early‐life experience (Balsam & Stevenson, 2020; Hedrick & Kortet, 2012; Niemelä, DiRienzo, et al., 2012; Niemelä, Vainikka, et al., 2012), social environment (Bailey et al., 2013; Fisher et al., 2019) and access to females (Brown et al., 2007; Montroy et al., 2016) on the expression of male aggression in insects, female crickets were removed from stock boxes once sex differences became apparent, approximately one month before adult eclosion, and maintained as above in separate containers. Male nymphs were isolated at the instar before sexual maturity and individually housed in cylindrical 100‐mL transparent plastic tubs and maintained twice weekly to replace food, water and shelter as above.

### 2.2 Experimental design

**FIGURE 1:**
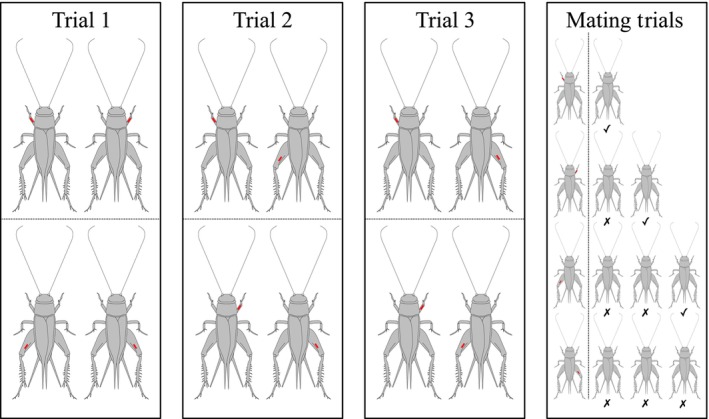
Illustration of the pairings of *Teleogryllus oceanicus* individuals in three successive trials, showing the marking system (red) identifying individuals within each replicate of four. The right panel illustrates the possible outcomes of mating trials. A focal male could mate with: the first female he was presented with (top row; high efficiency), the second female (second row; medium efficiency), the third female (third row; low efficiency), or no female in any of the three trials (bottom row; never mated).

Experimental males were haphazardly assigned to a replicate of four individuals. Each male in a replicate was identified with one of four unique markings, which we made by applying a dot using equal volumes of white acrylic paint (System 3 Original, ‘Titanium’) to one of their front or hind (right or left) femurs. All males were between 5 and 10 days of post‐adult eclosion at the start of their first trial, and we avoided experimental confounds by distributing all paired combinations evenly across trials (Figure 1).

#### 2.2.1 Measuring aggression

Each male was paired with each of the other three males in his replicate over three consecutive days, completing a set of three aggression trials per replicate where every male had interacted with every other male in the replicate (Figure 1). A total of 124 males were used. This design enabled us to analyse aggression that each male instigated and aggression that each male experienced. The separate analyses described below examining instigation and experience of aggression were thus performed on the same set of 124 males. Trials were conducted in 14‐L plastic containers under red light, at 24–26°C, during the crickets' night cycle. Trials were recorded using a camera (Nikon D3300 with Sigma 17–50 mm F2.8 EX DC OS HSM lens) above the centre of the arena.

Paired males were introduced into the container equidistant from the centre and its edges to ensure no perceived advantage in terms of starting territory. The side of the container on which a particular cricket started trials randomised across days, as was the order in which pairs within each replicate were trialled. After acclimating for 2 min under a 100‐mL clear plastic container, the containers were lifted and the trial began. Trials lasted 10 min. If a cricket attempted to escape, a sheet of glass was placed atop the container, allowing the camera to continue recording the activity beneath. At the end of each trial, males were returned to their containers with new food and water and the container was wiped down with 70% ethanol to remove residual scent cues. To score behavioural data, interactions were identified and timed in real time, with video recordings analysed in a randomised order to validate those observations.

We quantified aggression based on how many times aggressive behaviours occurred during a trial, their duration, and the identity of the male that instigated them. Some behaviours involved in aggressive contexts may be ambiguous because they are used in other contexts. To avoid the risk of misclassification and ensure that we scored aggression and no other investigative behaviours, we defined it as either mandibular contact or sparring, as these represent unambiguous expressions of aggressive behaviour. We required that a period of 5 s passed without any aggressive behaviour, before recording further aggressive behaviours as a separate bout. From these data, we quantified *instigated aggression* per trial and across all trials by recording the number of altercations instigated by a focal male, his total and average number of instigated aggressive behaviours, and the total and average duration of aggression that he instigated. Data for *experienced aggression* were the same values but applied to the cricket receiving aggression within each pairing. We defined the instigator as the male that made first contact with his mandibles or tarsi and note that a male could both instigate and experience aggression within a single trial due to the occurrence of more than one bout of behaviour. An experiment‐level aggression score was used to quantify the number of trials in which each male instigated or experienced aggression (ranging from 0 to 3). We also recorded whether any individual inflicted or experienced significant bodily harm (dismemberment, for example, *N* = 1).

#### 2.2.2 Mating trials

A subset of 96 of the original 124 males was further tested in mating trials. We were unable to test all 124 due to interruption by the first UK COVID‐19 lockdown in March 2020, which resulted in restricted access to laboratories. On the day following the final aggression trial, three virgin females per male were haphazardly selected and isolated in 100‐mL lidded tubs. All females were between 5 and 10 days of postadult eclosion to ensure sexual receptivity. One male was placed in one half of a 14‐L plastic container, and in the remaining half, a female haphazardly selected from those isolated. After a 2‐min acclimation period, isolation chambers were lifted, and the pair were permitted to freely interact. Trials lasted 20 min. We recorded whether the individuals successfully mated (i.e. mounting resulting in spermatophore transfer); whether either individual made an unsuccessful attempt to mate; and whether either individual was aggressive towards the other.

All crickets that successfully mated were not used in the experiment again. We also removed from subsequent trials any unmated females who had been in excessive contact with a male (i.e. if physical contact occurred between more than just their antennae, or if physical damage had been inflicted) due to the possibility of decreased physical condition or cuticular hydrocarbon (CHC) transfer. Unmated and unharmed females, and females that were initially isolated but not used, were returned to same‐sex stock boxes and not reused in trials for at least 1 week. Males that did not mate with the first female with whom they were paired were isolated in their containers for at least 1 h before being retrialled with a second female. Males were given a maximum of three females with whom to attempt mating (Figure 1). *Mating success* was a binary measure indicating whether or not a male successfully mated with a female during mating trials, regardless of how many opportunities he required. *Mating efficiency* was defined as the inverse of the number of females presented to a male before he successfully mated (i.e. only one female required equated to ‘High’ efficiency, two females to ‘Medium’ and three to ‘Low’—with a separate category for males who could be described as having ‘Never Mated’). Efficiency thus indicates the number of foregone mating opportunities, which could reflect the tendency to maximise potential reproductive success through efficient utilisation of resources, or the degree of male choosiness.

### 2.3 Analysis

Data were visually inspected and analysed using RStudio (R Core Team, 2022; RStudio Team, 2016); packages ‘gridExtra’ (Auguie, 2017), ‘lme4’ (Bates et al., 2015), ‘Matrix’ (Bates et al., 2022), and ‘tidyverse’ (Wickham et al., 2019). Expressions describing statistical models reported below are given in Table S1 (Equations 1–12).

#### 2.3.1 Variation and repeatability of male intrasexual aggression

Our analyses focussed on patterns of aggression instigated by focal males towards their interacting partners and aggression experienced by interacting partners. The standard method of estimating repeatability is to calculate the intraclass correlation coefficient (ICC) using an analysis of variance (ANOVA) (Lessells & Boag, 1987). However, this was not possible for our experiment due to the binary data structure and retrialling of individuals within replicates, precluding the statistical independence required for standard ANOVA. Pseudoreplication to produce an ICC would unacceptably skew inference, and the data transformations required for a parametric approach would also result in estimates of low confidence (Wu et al., 2012). This, plus the fact that evidence for significant repeatability, was not found using generalised linear models (GLMs) suited to the data structure (see below), made an ANOVA‐based approach to produce estimates using the ICC inappropriate.

We focussed on the subset of males that initiated or experienced aggression in at least one trial to evaluate the effect of focal and interacting partner identity on the total number of aggressive encounters instigated by the focal individual (which, in some cases, was more than once per trial), and the average duration for which the encounters lasted (with the trial‐level average per aggressive bout used to account for the fact that ‘encounter duration’ covaries with number of bouts). Data were analysed using, first, chi‐squared tests to ascertain whether the distribution of instigated and experienced aggression across trials departed from random expectation and, second, linear mixed‐effects models (LMMs) with log‐likelihood ratio tests (LRTs) including focal and interacting individual identities as random effects (Table S1, Equation 1). We examined the relative influence of focal and interacting individual identity as random effects on focal aggression by using an LRT to compare models with and without each random effect. The latter results should not be interpreted as precise effect sizes with associated statistical significance, but rather as indicative of the relative influence of direct (focal individual) effects on aggression compared with indirect (interacting individual) effects.

Finding little evidence for indirect effects of partner identity in the above analyses (see Section 3), we next examined the effect that experience of aggression in previous trials had on an individual's tendency to instigate or experience aggression in later trials. First, a chi‐squared test was used to examine the distribution of aggressive instigations across trials. Second, generalised linear mixed‐effects models (GLMM) with a binomial error structure were used to model the overdispersed, zero‐inflated data of the binary aggression measures (Table S1, Equations 2–5). Separate models were run for responses of instigating or experiencing aggression in trials 2 and 3, for four models total. Identity of the focal individual was included in each model as a random effect and LRTs were performed with single‐term deletions to assess significance of the binary variables describing aggression instigated and experienced in previous trials, plus their interactions.

#### 2.3.2 Male intrasexual aggression and mating success

Finding little evidence for repeatability or partner effects on either instigated or experienced male aggression, the next set of analyses tested whether focal male aggression during intrasexual aggression trials was associated with mating outcomes during subsequent intersexual mating trials. We modelled (Table S1, Equation 8) the effects of experiment‐level aggression scores (0–3, as described above) and total number of aggressive instigations on mating success (1/0) using binary logistic regressions, and we examined the relationship of experiment‐level aggression scores (0–3 as above) and total number of aggressive instigations on mating efficiency using Spearman's rank correlations. We also tested whether the duration of instigated aggression affected mating success (1/0) and efficiency, examining both the total and average duration spent actively sparring with other males (Table S1, Equation 9). To investigate whether experiencing aggression affected male mating success, we performed the same analyses as above but with factors reflecting experienced aggression in place of instigated aggression (Table S1, Equation 10).

#### 2.3.3 Male‐instigated intersexual aggression and mating success

Over the course of the mating trials, we observed that some males instigated aggression towards their female partners. To explore consequences of this behaviour, we examined its relationship to male mating success. First, we examined factors that might explain the expression of intersexual aggression by modelling the number of trials in which males previously instigated or experienced intrasexual aggression, and the total and average duration for which males were aggressive using binary logistic regression with male expression of intersexual aggression (1/0) as the dependent variable (Table S1, Equation 11). Next, we examined how male intersexual aggression (1/0) was related to his mating success (1/0) using a chi‐squared test, and how male intersexual aggression (1/0) was related to his mating using a Wilcoxon rank sum test. The number of females towards whom he displayed aggression (0–3) was also tested as an explanatory variable for these measures using binary logistic regression and GLM, respectively (Table S1, Equation 12).

## 3 RESULTS

### 3.1 Variation and repeatability of male intrasexual aggression

In total, 186 paired aggression trials were run. These trials comprised 31 replicates of four individuals each, and for each replicate, we ran two trials on each of 3 days representing the two unique pairings available. Of the 124 males used for trials, 89 (71.9%) instigated aggression towards, and 90 (72.6%) experienced it from, at least one of their interacting male partners. Only nine (7.3%) males instigated, and eight (6.5%) experienced, aggressive bouts in all three trials. Whether or not a male instigated aggression varied across the three successive trials (chi‐square test: $\chi_{3}^{2}$ = 18.816, *N* = 124, *p* < .001), as did whether aggression was experienced (chi‐square test: $\chi_{3}^{2}$ = 18.581, *N* = 124, *p <* .001); individual males were most likely to instigate/experience aggression only once, and the number of aggressive instances tended to decrease across subsequent trials. In GLMMs examining focal versus partner effects, neither focal nor interacting male identity within dyads predicted whether males instigated aggression (log‐likelihood ratio tests: focal: $\chi_{5}^{2}$ = 0.260, *N* = 124, *p* = .130; interacting: $\chi_{5}^{2}$ = 0.808, *N* = 124, *p* = .404). In this model, however, the variance explained by the focal random effect (0.277) was an order of magnitude higher than that associated with the interacting random effect (0.057).

Similarly, male identity did not predict the duration of aggressive encounters observed (log‐likelihood ratio tests: focal: $\chi_{5}^{2}$ < 0.001, *N* = 124, *p* = .500; interacting: $\chi_{5}^{2}$ = 0.980, *N* = 124, *p* = .155). Despite a general increase in the instigation of aggression over the course of the three trials (likelihood ratio test: $\chi_{1}^{2}$ = 6.777, *p* = .009), we found no evidence for an influence of previous aggressive interactions on the instigation or experience of aggression in later trials (GLMMs: all *p* ≥ .083; full statistical results in Table S2).

### 3.2 Male intrasexual aggression and mating success

Of the subset of *N* = 96 males that entered mating trials, 80 (83.3%) eventually mated. Neither the number of trials (0–3) in which males instigated aggression nor the total number of aggressive instigations predicted mating success (binary logistic regressions, respectively: *z*_1,95_ = −0.514, *p* = .607; *z*_1,95_ = −0.441, *p* = .660) or efficiency (Spearman's rank correlations, respectively: *r*_s_ = .089, *N* = 96, *p* = .386; and *r*_s_ = .048, *N* = 96, *p* = .640). The duration for which males expressed aggressive behaviour was also unrelated to mating success (binary logistic regressions for total duration of aggression: *z*_95_ = −0.079, *p* = .937; and average duration of aggression: *z*_95_ = 0.147, *p* = .883). Similarly, mating efficiency was unrelated to total or average duration of male aggression (Spearman's rank correlations, respectively: *r*_s_ = .074, *N* = 96, *p* = .477, and *r*_s_ = .080, *N* = 96, *p* = .438).

**FIGURE 2:**
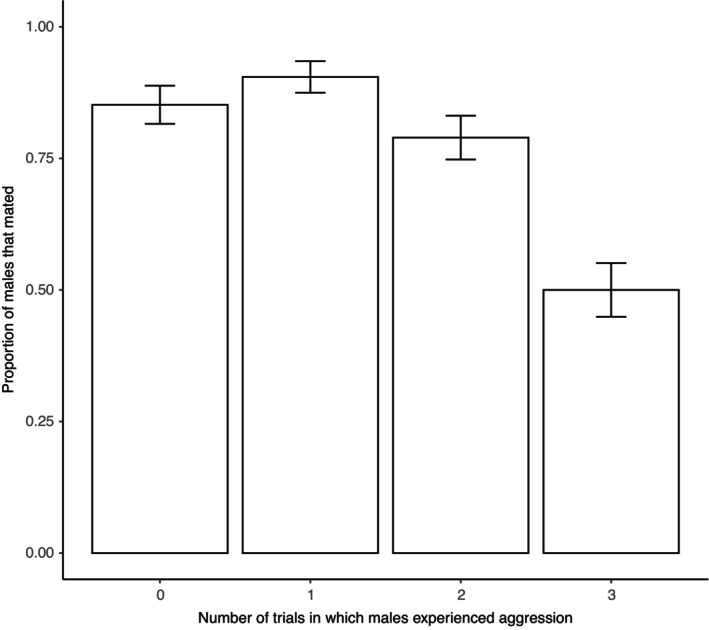
Relationship between *Teleogryllus oceanicus* male mating success and the number of trials in which males experienced aggression. Error bars indicate standard error of the respective proportions.

Males who consistently experienced aggression from other males in previous trials had lower mating success (50.0% mated) than males who consistently did not experience any aggression (85.2% mated) (Figure 2) (binary logistic regression: *z*_95_ = −1.979, *p* = .048). Prior experience of aggression (in terms of both consistency and total number of experiences) was also associated with mating efficiency (Spearman's rank correlation: *r*_s_ = .247, *N* = 96, *p* = .016, and *r*_s_ = .247, *N* = 96, *p* = .015, respectively).

The total number of times that males experienced aggression had no significant effect on mating success (binary logistic regression: *z*_95_ = −1.914, *p* = .056), but negatively affected mating efficiency (Spearman's rank correlation: *r*_s_ = −.227, *N* = 96, *p* = .026). Conversely, neither the total nor the average duration for which males experienced aggression in previous trials predicted mating success (binary logistic regressions, respectively: *z*_95_ = −0.569, *p* = .569; and *z*_95_ = −0.596, *p* = .551) or efficiency (Spearman's rank correlations, respectively: *r*_s_ = −.156, *N* = 96, *p* = .129, and *r*_s_ = −.162, *N* = 96, *p* = .114).

### 3.3 Male‐instigated intersexual aggression and mating success

**FIGURE 3:**
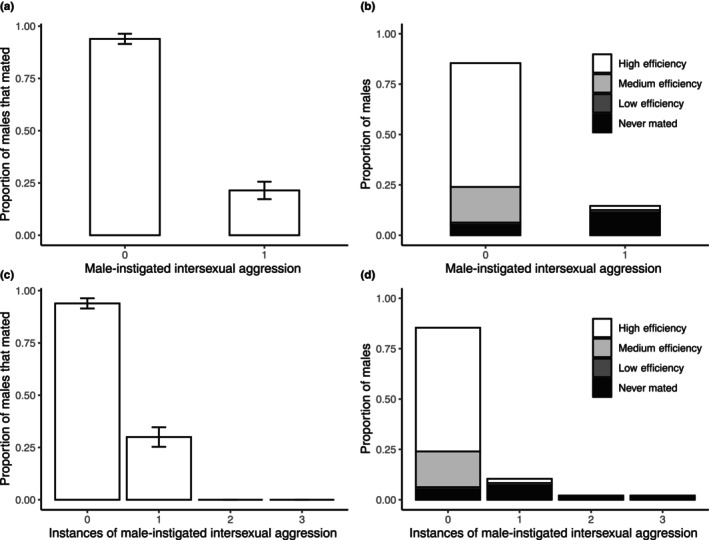
The relationship between intersexual aggression and male mating success and efficiency. (a) The proportion of males that did (1) or did not (0) successfully mate after instigating intersexual aggression. Error bars indicate proportional standard error. (b) Mating efficiency of males that did (1) or did not (0) instigate intersexual aggression, showing proportions of males across all trials with high, medium, or low mating efficiency, or no mating (shading). (c) The proportion of males that successfully mated depending on the number of trials in which they instigated intersexual aggression. Error bars indicate proportional standard error. (d) Mating efficiency of males depending on the number of trials in which they instigated intersexual aggression, showing proportions of males across all trials with high, medium, or low mating efficiency, or no mating (shading). No male instigating intersexual aggression in more than one trial successfully mated. Note that proportions in panels (a) and (c) do not sum to 1 because they illustrate proportions of mated males within each category separately, whereas proportions in panels (b) and (d) sum to 1 because they illustrate proportions of males with different mating efficiencies across all trials (*N* = 96). There were extremely few occurrences (*N* = 1) of ‘low efficiency’ mating, so these are difficult to discern in panels b and d; low efficiency matings only occurred when males instigated one intersexual aggressive bout.

Over the course of the mating experiment, we made the unexpected observation that 14 (14.6%) of males instigated intersexual aggression towards one or more of their female partners, although only two of these appeared to do so consistently across mating trials with multiple females. Of the 82 males who did not express intersexual aggression, 77 (93.9%) successfully mated. However, only three out of 10 (33.3%) males who were aggressive towards one female successfully mated. No male who expressed aggressive behaviour towards more than one female achieved a successful mating (*N* = 4). Males' past experience of instigating or experiencing aggression did not affect their propensity for behaving aggressively towards females (binary logistic regressions, respectively: *t*_95_ = 0.597, *p* = .551; and *t*_95_ = 1.832, *p* = .067), nor did the total or average duration for which they were aggressive (binary logistic regressions, respectively: *t*_95_ = −0.777, *p* = .437; and *t*_95_ = −1.018, *p* = .309). However, engaging in any intersexual aggression at all during mating trials decreased both male mating success (chi‐square test: $\chi_{1}^{2}$ = 40.156, *N* = 96, *p* < .001) (Figure 3a) and efficiency (Wilcoxon rank‐sum test: *W* = 979, *p* < .001) (Figure 3b). The number of females towards whom a male instigated intersexual aggression similarly affected mating success (binary logistic regression: *t*_95_ = −4.432, *p* < .001) (Figure 3c) and efficiency (GLM: *t*_95_ = −5.378, *p* < .001) (Figure 3d).

## 4 DISCUSSION

The only consistent characteristic of male aggression in this study was its inconsistency. Focal male identity explained more variance than partner effects, the latter being negligible, and we found little evidence that prior experience of aggression influenced the expression or experience of aggression in subsequent male–male encounters. Despite this apparent unpredictability, aggression was associated with male mating success in two ways. First, male experience of aggression in previous male–male trials was associated with lower mating success, providing evidence for fitness consequences of being exposed to more aggressive interacting partners, which is known to inflict physical damage (Judge & Bonanno, 2008; Kuriwada, 2017). Second, male aggression towards females was associated with lower mating success, which is consistent with nonadaptive aggressive spillover or mistaken identity. The consequences of such spillover have not often been considered outside the context of sexual cannibalism, but are consistent with observations of other male–male behaviours in *T. oceanicus*, such as same‐sex sexual behaviour (Bailey & French, 2012; Rayner & Bailey, 2019).

It was unsurprising that both the expression of aggression and the tendency to receive it from others showed no strong signal of repeatability, although we did find that partner effects were an order of magnitude weaker than direct effects. This implies that more variation in the expression of aggression originates with the individual instigating it than with characteristics of their interacting partner. One interpretation of our results is that flexibility in aggression may provide fitness benefits, but we have not discovered the causal factors underlying such flexibility. It is possible that contingency and stochasticity play a large role in determining which partner initiates aggression first or most frequently, such as energy immediately available for metabolic activity or other chance factors. The surprisingly frequent expression of intersexual aggression that we observed (13.5% of males) had the most clear negative impact on male mating success. While this finding is intuitively logical, it has not often been reported and it raises the question as to why intersexual aggression, if so detrimental to mating success, is expressed at all.

Taken together, the intersexual aggression we observed is consistent with aggressive spillover or mistaken identity. Spillover occurs when a behaviour that is adaptive in one context—in this case, male–male aggression—is expressed in another in which it is not, such as during a mating encounter (Arnqvist & Henriksson, 1997; Duckworth, 2006; Johnson & Sih, 2005; Wijnhorst, 2017). Spillover as it was originally described in the context of sexual cannibalism is an explanation at the level of evolutionary function, not at the level of mechanisms of individual behavioural expression, because it refers to the non‐adaptive expression of a behaviour in one context which happens because the behaviour evolved for an adaptive function in another context and context‐dependent expression is not complete. In this sense, it is akin to an evolutionary by‐product. While it is not unheard of for males to be observed directing aggressive behaviours towards females (Akin, 1998; Connor et al., 2005; Huffard et al., 2010), it is a behaviour that, in terms of optimising fitness, seems counterintuitive. Intersexual aggression has been particularly well‐documented in promiscuous primate species employing sexual coercion such as chimpanzees (*Pan troglodytes*), in which it has been hypothesised that male‐instigated intersexual aggression evolved as a form of sexual coercion to minimise paternity confusion (Feldblum et al., 2014; Muller et al., 2007). However, many other studies have suggested that this behaviour is not adaptive (Fruth & Hohmann, 2003; Stumpf & Boesch, 2010). Apart from research on humans and primates, little is known about the relationship of intersexual aggression and mating strategy (though see Felice & Dukas, 2022 who found a later benefit for females in *Drosophila melanogaster*). Unlike primates, crickets are not highly social, and mating is under the control of females who only mount males with whom they choose to mate. In our study, males that expressed intersexual aggression experienced an approximately 60% reduction in mating success. Doing so on more than one occasion was associated with no mating at all.

It is important to note that spillover explanations require neither behavioural repeatability, which we did not find, nor a phenotypic correlation between behaviours across contexts, which we also did not find. A spillover explanation *does* require showing nonadaptive expression of behaviour in a context separate to that in which its adaptive function evolved, which our results demonstrate. Another assumption of aggressive spillover explanations is that behaviour has not evolved to be sufficiently flexible or responsive for adaptive, context‐dependent expression. Yet in male–male trials, instigation of aggression appeared to be highly flexible, and males that were more aggressive in male–male contests were no more or less likely to be aggressive towards females. The expression of intersexual aggression could also result from a low acceptance threshold for identifying competitors (Reeve, 1989). This means that some males might maladaptively direct aggressive behaviours towards females. Intersexual aggression is consistent with Reeve's (1989) acceptance threshold theory, which suggests that imperfect conspecific recognition (either of sex or of kin) may be adaptive if the fitness cost of acting on mistaken identity is lower than that of inaction. This idea has been used to explain other behavioural phenotypes that appear to be nonadaptive, namely same‐sex sexual behaviour (SSB) (Bailey & Zuk, 2009; Engel et al., 2015; Lerch & Servedio, 2021; Sales et al., 2018).

We observed intersexual aggression at a level comparable to that of SSB in the same species (16%–26%) (Bailey & French, 2012; Rayner & Bailey, 2019). The latter studies suggested a link between the pre‐trial social environment and subjects' perception of the relative costs associated with mistaken identity of either sex. Our study differed in that males were kept in complete social isolation prior to reaching sexual maturity and had never encountered an adult female. This was to ensure such mistaken identity could not occur: male and female crickets recognise one another innately, without experience, and through multiple signal channels; sexual dimorphism in acoustic mating signals and cuticular hydrocarbon (CHC) pheromone signals are unambiguous, prominent, and extremely well‐documented in field crickets (Tregenza & Wedell, 1997). CHCs are specifically known to be sexually dimorphic in *T. oceanicus* (Moran et al., 2019; Pascoal et al., 2020; Thomas & Simmons, 2008, 2010), so intersexual aggressive behaviour is not parsimoniously explained simply by the phenomenon of mistaken identity. These conditions reflect realistic conditions in the wild, where crickets may be spaced far apart (>1 m) and have low encounter rates. What isolated crickets in our experiment might have perceived, therefore, is a sparse social environment. By the time a male encountered a female in our study, he had for his entire adult life only encountered other males in aggression trials. This lack of social experience could have altered his perception of potential mating opportunities, stimulating greater investment in aggression to cope with an environment of apparent high male–male competition, and thus increasing the likelihood of expressing the behaviour in a nonadaptive context. Conversely, a male who had previously encountered females might invest more heavily in courtship due to the perception of mate availability.

The lack of evidence for strong intra‐individual repeatability of aggression does not exclude the possibility that it is repeatable in some other context, and it contrasts with findings in another cricket species, *Gryllus bimaculatus* (e.g. Santostefano et al., 2017a). Nevertheless, the labile expression we observed has implications for understanding the ultimate evolutionary origin and persistence of aggression. For example, one explanation for our findings is that there is little underlying genetic variation for aggression (Olzer et al., 2019). This would obviously constrain its evolution. Alternatively, it may be adaptive in the context of intense male–male scramble competition to deploy agonistic behaviours in a more flexible manner (Wright et al., 2010). Our findings favour the latter explanation and are consistent with previous findings about the environmental sensitivity of aggression in related species. For example, there is ample evidence that male crickets instigate aggression to establish dominance (Bunting & Hedrick, 2018; Thomas & Simmons, 2009). Discriminate aggression towards individuals may also reflect characteristics of phenotypic incompatibility (Santostefano et al., 2016, 2017b). It is commonly assumed that aggressive behaviour is adaptive, an intuitive conclusion in field crickets due to its frequent and widespread nature (Alexander, 1962; Rillich et al., 2019; Stevenson et al., 2005). However, intersexual aggression is associated with significant negative consequences, so selection should theoretically favour accurate perception and response to the prevailing social environment.

Our results highlight the need to consider behavioural phenotypes integrated across all contexts to establish their total fitness consequences (Bell, 2006; Duckworth, 2006). In particular, the precision with which individuals can facultatively adjust behaviour based on social context and interacting individuals warrants further research. The intersexual aggression that we found suggests that it will be important to establish physiological and sensory mechanisms responsible for the limitations on how quickly or adaptively an individual can adjust their behavioural expression to new interacting partners, even when they are sexually receptive individuals of the opposite sex. It would be informative to test the limits of that environmental sensitivity in future studies, as our results clearly indicate it is not complete. Our findings also suggest it may be profitable to consider a broader problem, which is that individuals may vary in their capacity to flexibly respond to prevailing conditions for traits such as aggression. Put another way, it is uncertain whether flexibility itself is repeatable at the intra‐individual level. Answering this question is a technically daunting challenge, but would ultimately inform our understanding not of how individual behaviours evolve, but how the *responsiveness* of behaviour to stimuli—arguably the essence of behaviour itself (Levitis et al., 2009) – evolves.

## AUTHOR CONTRIBUTIONS

**Eleanor K. Tinsley:** Conceptualization (equal); data curation (lead); formal analysis (lead); investigation (lead); methodology (lead); project administration (lead); software (lead); validation (lead); visualization (lead); writing – original draft (lead); writing – review and editing (equal). **Nathan W. Bailey:** Conceptualization (equal); funding acquisition (lead); methodology (supporting); resources (lead); supervision (lead); writing – review and editing (equal).

## FUNDING INFORMATION

This work was supported by the UK Natural Environment Research Council (NE/L011255/1 and NE/T000619/1).

## Supporting information


Data S1

Table S1

## Data Availability

All data, metadata and explanations of analyses and R code are publicly available at https://github.com/ektinsley/Tinsley‐Bailey.
