# Correction to “Intrasexual aggression reduces mating success in field crickets”

**DOI:** 10.1002/ece3.10663

**Published:** 2023-10-24

Tinsley, E. K**.** & Bailey, N. W. (2023). Intrasexual aggression reduces mating success in field crickets. *Ecology and Evolution*, **13**(10): e10557. doi: **10.1002/ece3.10557**

The title of this article “Intrasexual aggression reduces mating success in field crickets” is incorrect. This should read: “Intersexual aggression reduces mating success in field crickets”.

We apologize for this error.

