# Supplementary material for: Intrasexual aggression reduces mating success in field crickets
Source: Ecol Evol. 2023 Oct 1;13(10):e10557. doi: 10.1002/ece3.10557 (PMC10542478; doi:10.1002/ece3.10557)
Supplement: Supplementary file 1 — Data S1 [file ECE3-13-e10557-s001.zip › Data and R Code/Tinsley & Bailey - R Script.docx]

# Intrasexual aggression reduces mating success in field crickets

# Eleanor K. Tinsley - ORCiD 0000-0001-6779-5272

# R Script

# Friday 25th August 2023

### Packages and Data ######################

rm(list=(ls()))

# R Studio Version:

RStudio.Version()

#R version 4.2.2 (2022-10-31 ucrt) -- "Innocent and Trusting"

#$mode

#[1] "desktop"

#$version

#[1] ‘2023.6.0.421’

#$long_version

#[1] "2023.06.0+421"

#$release_name

#[1] "Mountain Hydrangea"

#citation()

# Analyses Libraries:

library("gridExtra")

library(lme4)

library(Matrix)

library(tidyverse)

# Package Audit Libraries:

library(devtools)

library("NCmisc")

# Citations:

citation("gridExtra")

citation("lme4")

citation("Matrix")

citation("tidyverse")

# Package Audit:

# Functions used in each package (base R):

library("NCmisc")

list.functions.in.file("Tinsley & Bailey - R Script.R")

#character(0):

# "aes", "element_blank", "gather", "geom_col", "geom_errorbar", "ggplot",

# "glmer", "grid.arrange", "lmer", "rename", "scale_fill_manual",

# "scale_y_continuous", "stale_package_check", "theme", "theme_classic",

# "xlab", "ylab"

#package:base:

# "c", "colnames", "factor", "library", "ls", "print", "rm", "subset",

# "summary", "table"

#package:devtools:

# "install_github"

#package:NCmisc:

# "list.functions.in.file"

#package:stats:

# "anova", "chisq.test", "cor.test", "glm", "logLik", "na.omit", "pchisq",

# "wilcox.test"

#package:utils:

# "citation", "read.csv", "View", "write.csv"

#$`tools:rstudio`

#[1] "RStudio.Version"

# Functions used in each package (loaded libraries):

library(devtools)

install_github("MichaelChirico/funchir")

funchir::stale_package_check('Tinsley & Bailey - R Script.R')

#package:Matrix:

# print, summary

#package:lme4:

# glmer, lmer

#package:ggplot2:

# aes, element_blank, geom_col, geom_errorbar, ggplot, scale_fill_manual,

# scale_y_continuous, theme, theme_classic, xlab, ylab

#package:gridExtra:

# grid.arrange

#package:NCmisc:

# list.functions.in.file

#package:devtools:

# install_github

#Data:

agg=read.csv("./R Data/Aggression.csv",header=TRUE)

aggmat=read.csv(file="./R Data/AggMat.csv",header=TRUE)

agg_removed=read.csv(file="./R Data/AggressiveOnly.csv",header=TRUE)

cs=read.csv(file="./R Data/Chi-Sq.csv",header=TRUE)

csEC=read.csv(file="./R Data/csEC.csv",header=TRUE)

csECna=read.csv(file="./R Data/csECna.csv",header=TRUE)

csER=read.csv(file="./R Data/csER.csv",header=TRUE)

csIC=read.csv(file="./R Data/csIC.csv",header=TRUE)

csICna=read.csv(file="./R Data/csICna.csv",header=TRUE)

csIR=read.csv(file="./R Data/csIR.csv",header=TRUE)

figs=read.csv(file="./R Data/Figs.csv",header=TRUE)

inds=read.csv(file="./R Data/Individuals.csv",header=TRUE)

matedonly=read.csv(file="./R Data/MatedOnly.csv",header=TRUE)

pairs=read.csv(file="./R Data/Pairings.csv",header=TRUE)

### Intrasexual Aggression - Repeatability ###################################

# (i) Variation and repeatability of male intrasexual aggression

# (Analyses 1-8)

# 1) Lines 204-205, 255

#Test: Sum of binary instigated aggression across trials (0-3)

# against random expectation

#Model: Chi-square

#Result: χ23 = 18.816, N = 124, p < 0.001 ***

# R Script:

csIC <- csIC %>% select(Cat:Obs)

View(csIC)

table(csIC$Cat, csIC$Obs)

csqIC <- chisq.test(table(csIC$Cat, csIC$Obs))

csqIC

subsetcsIC <- subset(csIC, select=c(Cat, Obs))

View(subsetcsIC)

csICna <- na.omit(subsetcsIC)

View(csICna)

table(csICna$Cat, csICna$Obs)

csqICna <- chisq.test(table(csICna$Cat, csICna$Obs))

csqICna

View(csICna)

table(csICna$Cat, csICna$Obs)

csq1 <- chisq.test(table(csICna$Cat, csICna$Obs))

csq1

#X-squared = 18.816, df = 3, p-value = 0.0007712 ***

# 2) Lines 204-205, 256

#Test: Sum of binary expressed aggression across trials (0-3)

# against random expectation

#Model: Chi-square

#Result: χ23 = 18.581, N = 124, p < 0.001 ***

# R Script:

csEC <- csEC %>% select(Cat,Obs)

View(csEC)

table(csEC$Cat, csEC$Obs)

csqEC <- chisq.test(table(csEC$Cat, csEC$Obs))

csqEC

subsetcsEC <- subset(csEC, select=c(Cat, Obs))

View(subsetcsEC)

csECna <- na.omit(subsetcsEC)

View(csECna)

table(csECna$Cat, csECna$Obs)

csqECna <- chisq.test(table(csECna$Cat, csECna$Obs))

csqECna

table(csECna$Cat, csECna$Obs)

csq2 <- chisq.test(table(csECna$Cat, csECna$Obs))

csq2

#X-squared = 18.581, df = 3, p-value = 0.0003338 ***

# 3) Lines 205-209, 260

#Test: Focal partner identity against aggression (1/0)

#Model: Linear mixed-effect model (LMM) with log-likelihood ratio test (LRT)

#Result: χ25 = 0.260, N = 124, p = 0.130

#R Script:

library(lme4)

colnames(inds)[1]<-"Focal"

m1<-glmer(Agg~Trial+(1|Focal)+(1|Interacting),family=binomial,data=inds)

mf<-glmer(Agg~Trial+(1|Focal),family=binomial,data=inds)

mi<-glmer(Agg~Trial+(1|Interacting),family=binomial,data=inds)

summary(m1)

#Random effects:

# Groups Name Variance Std.Dev.

#Interacting (Intercept) 0.05661 0.2379

#Focal (Intercept) 0.27699 0.5263

#Focal:

anova(mi,m1)

# npar AIC BIC logLik deviance Chisq Df Pr(>Chisq)

#m2 3 491.14 502.89 -242.57 485.14

#m1 4 491.87 507.55 -241.94 483.87 1.2676 1 0.2602

pchisq(2*(logLik(m1)-logLik(mi)),df=1,lower.tail=FALSE)/2

#'log Lik.' 0.1301068 (df=4)

# 4) Lines 205-209, 260-261

#Test: Interacting partner identity against aggression (1/0)

#Model: Linear mixed-effect model (LMM) with log-likelihood ratio test (LRT)

#Result: χ25 = 0.808, N = 124, p = 0.404

#R Script:

colnames(inds)[1]<-"Focal"

m1<-glmer(Agg~Trial+(1|Focal)+(1|Interacting),family=binomial,data=inds)

mf<-glmer(Agg~Trial+(1|Focal),family=binomial,data=inds)

mi<-glmer(Agg~Trial+(1|Interacting),family=binomial,data=inds)

summary(m1)

#Random effects:

# Groups Name Variance Std.Dev.

#Interacting (Intercept) 0.05661 0.2379

#Focal (Intercept) 0.27699 0.5263

# Interacting:

anova(mf,m1)

# npar AIC BIC logLik deviance Chisq Df Pr(>Chisq)

#m3 3 489.93 501.69 -241.96 483.93

#m1 4 491.87 507.55 -241.94 483.87 0.059 1 0.8081

pchisq(2*(logLik(m1)-logLik(mf)),df=1,lower.tail=FALSE)/2

#'log Lik.' 0.404032 (df=4)

# 5) Lines 205-209, 264

#Test: Focal partner identity against total aggressive duration

#Model: Linear mixed-effect model (LMM) with log-likelihood ratio test (LRT)

#Result: χ25 <0.001, N = 124, p = 0.500

#R Script:

colnames(inds)[1]<-"Focal"

subset_inds <- subset(inds, X.I > 0)

View(subset_inds)

duration <- lmer(Dur~Trial+(1|Focal)+(1|Interacting),data=subset_inds)

summary(duration)

#Random effects:

#Groups Name Variance Std.Dev.

#Interacting (Intercept) 3.705e+01 6.08697

#Focal (Intercept) 1.688e-04 0.01299

#Residual 1.773e+02 13.31615

#Number of obs: 135, groups: Interacting, 90; Focal, 88

reduceddurationf <- lmer(Dur~Trial+(1|Interacting),data=subset_inds)

reduceddurationf2 <- glmer(Dur~Trial+(1|Interacting),data=subset_inds)

summary(reduceddurationf2)

#REML criterion at convergence: 1099.1

#Scaled residuals:

# Min 1Q Median 3Q Max

#-1.3246 -0.7214 -0.1832 0.4542 2.8586

#Random effects:

#Groups Name Variance Std.Dev.

#Interacting (Intercept) 37.05 6.087

#Residual 177.32 13.316

#Number of obs: 135, groups: Interacting, 90

#Fixed effects:

# Estimate Std. Error t value

#(Intercept) 16.698 3.495 4.777

#Trial 1.126 1.530 0.736

#Correlation of Fixed Effects:

#(Intr)

#Trial -0.924

anova(reduceddurationf,duration)

#refitting model(s) with ML (instead of REML)

# reduceddurationf: Dur ~ Trial + (1 | Interacting)

#duration: Dur ~ Trial + (1 | Focal) + (1 | Interacting)

# Df AIC BIC logLik deviance Chisq Chi Df Pr(>Chisq)

#reduceddurationf 4 1112.2 1123.8 -552.1 1104.2

#duration 5 1114.2 1128.7 -552.1 1104.2 0 1 1

#Focal removed loglik:

lrt5 <- pchisq(2*(logLik(duration)-logLik(reduceddurationf)),df=1,

lower.tail=FALSE)/2

lrt5

#'log Lik.' 0.5 (df=5)

# 6) Lines 205-209, 264

#Test: Interacting partner identity against total aggressive duration

#Model: Linear mixed-effect model (LMM) with log-likelihood ratio test (LRT)

#Result: χ25 = 0.980, N = 124, p = 0.155

#R Script:

duration <- lmer(Dur~Trial+(1|Focal)+(1|Interacting),data=subset_inds)

reduceddurationi <- lmer(Dur~Trial+(1|Focal),data=subset_inds)

reduceddurationi2 <- glmer(Dur~Trial+(1|Focal),data=subset_inds)

summary(reduceddurationi2)

#REML criterion at convergence: 1100.1

#Scaled residuals:

# Min 1Q Median 3Q Max

#-1.2739 -0.7975 -0.1997 0.5437 2.8769

#Random effects:

# Groups Name Variance Std.Dev.

#Focal (Intercept) 8.625 2.937

#Residual 205.008 14.318

#Number of obs: 135, groups: Focal, 88

#Fixed effects:

# Estimate Std. Error t value

#(Intercept) 17.2681 3.5189 4.907

#Trial 0.9038 1.5572 0.580

#Correlation of Fixed Effects:

#(Intr)

#Trial -0.932

anova(reduceddurationi,duration)

#refitting model(s) with ML (instead of REML)

# reduceddurationi: Dur ~ Trial + (1 | Focal)

#duration: Dur ~ Trial + (1 | Focal) + (1 | Interacting)

# Df AIC BIC logLik deviance Chisq Chi Df Pr(>Chisq)

#reduceddurationi 4 1113.2 1124.8 -552.59 1105.2

#duration 5 1114.2 1128.7 -552.10 1104.2 0.9795 1 0.3223

#Interacting removed loglik:

lrt6 <- pchisq(2*(logLik(duration)-logLik(reduceddurationi)),df=1,

lower.tail=FALSE)/2

lrt6

#'log Lik.' 0.1551176 (df=5)

# 7) Lines 215-216, 265-266

#Test: Number of aggressive instigations against trial number (1-3)

##Model: Likelihood ratio test

#Result: χ21 = 6.777, p = 0.009 **

# R Script:

glm7<-glm(X.I~Trial,data=inds,family="poisson")

glm7.null<-glm(X.I~1,data=inds,family="poisson")

lrt7 <- anova(glm7,glm7.null,test="Chisq")

lrt7

#Results:

# Model 1: X.I ~ Trial

#Model 2: X.I ~ 1

#Resid. Df Resid. Dev Df Deviance Pr(>Chi)

#1 370 407.64

#2 371 414.42 -1 -6.7767 0.009235 **

# 8) Lines 216-222, 267-268

#Test: Instigated / experienced aggression in trials 2 & 3 against instigated /

# experienced aggression in trials 1 and 2 where appropriate, with focal ID

# included as a random effect

#Model: Generalised linear mixed-effects models (GLMM) + single-term deletions

#Result: All p > 0.08; full statistical results in Supplementary Table S2

#R Script:

glmm8 <- glmer(E3~I1*I2*E1*E2+(1|ID),family=binomial,data=agg)

summary(glmm8)

#Fixed effects:

#Estimate Std. Error z value Pr(>|z|)

#(Intercept) -0.0953 0.4371 -0.218 0.8274

#I1 0.6549 0.7691 0.852 0.3945

#I2 -1.3710 0.7915 -1.732 0.0832 .

#E1 -1.8506 1.1710 -1.580 0.1140

#E2 -0.2614 0.6596 -0.396 0.6919

#I1:I2 0.1183 1.2223 0.097 0.9229

#I1:E1 1.2910 1.9341 0.668 0.5044

#I2:E1 2.4006 1.5829 1.517 0.1294

#I1:E2 -0.7037 1.2919 -0.545 0.5859

#I2:E2 20.4608 788.7164 0.026 0.9793

#E1:E2 1.5141 1.4489 1.045 0.2960

#I1:I2:E1 -19.8971 1097.6763 -0.018 0.9855

#I1:I2:E2 0.2095 1182.5813 0.000 0.9999

#I1:E1:E2 0.1441 2.6092 0.055 0.9560

#I2:E1:E2 -21.4904 788.7164 -0.027 0.9783

#I1:I2:E1:E2 19.5694 1752.3291 0.011 0.9911

### Intrasexual Aggression- Mating Success ################

# (ii) Male intrasexual aggression and mating success

# (Analyses 9-25)

# 9) Lines 226-229, 272-273

#Test: Experiment-level aggression (0-3) against mating success (1/0)

#Model: Binary logistic regression

#Result: z1,95 = -0.514, p = 0.607

#R Script:

blr9 <- glm(Mated~IC, data = aggmat, family = "binomial")

summary(blr9)

#Results:

# Estimate Std. Error z value Pr(>|z|)

#(Intercept) 1.7853 0.4481 3.984 6.78e-05 ***

#IC -0.1564 0.3041 -0.514 0.607

# 10) Lines 226-229, 272-273

#Test: Total instigations of aggression against mating success (1/0)

#Model: Binary logistic regression

#Result: z1,95 = -0.441, p = 0.660

#R Script:

blr10 <- glm(Mated~X.I, data = aggmat, family = "binomial")

summary(blr10)

#Results:

#Estimate Std. Error z value Pr(>|z|)

#(Intercept) 1.73730 0.40678 4.271 1.95e-05 ***

#X.I -0.08642 0.19614 -0.441 0.66

# 11) Lines 229-230, 273-274

#Test: Experiment-level aggression (0-3) against mating efficiency

#Model: Spearman rank correlation coefficient

#Result: rs = 0.089, N = 96, p = 0.386

#R Script:

srcc11 <- cor.test(aggmat$F.,aggmat$IC,method='spearman')

srcc11

#Results:

#S = 134260, rho = 0.08939278, p-value = 0.3864

# 12) Lines 229-230, 273-274

#Test: Total instigations of aggression against mating efficiency

##Model: Spearman rank correlation coefficient

#Result rs = 0.048, N = 96, p = 0.640

#R Script:

srcc12 <- cor.test(aggmat$F.,aggmat$X.I,method='spearman')

srcc12

#Results:

#S = 140322, rho = 0.04827882, p-value = 0.6404

# 13) Lines 230-233, 275-276

#Test: Total duration of instigated aggression against mating success (1/0)

#Model: Binary logistic regression

#Result: z95 = -0.079, p = 0.937

#R Script:

blr13 <- glm(Mated~TDur, data = aggmat, family = "binomial")

summary(blr13)

#Results:

#Estimate Std. Error z value Pr(>|z|)

#(Intercept) 1.628065 0.362382 4.493 7.03e-06 ***

#TDur -0.001033 0.013058 -0.079 0.937

# 14) Lines 230-233, 275-277

#Test: Average duration of instigated aggression against mating success (1/0)

#Model: Binary logistic regression

#Result: z95 = 0.147, p = 0.883

#R Script:

blr14 <- glm(Mated~AvDur, data = aggmat, family = "binomial")

summary(blr14)

#Results:

#Estimate Std. Error z value Pr(>|z|)

#(Intercept) 1.569686 0.382045 4.109 3.98e-05 ***

#AvDur 0.004421 0.030043 0.147 0.883

# 15) Lines 230-233, 278

#Test: Total duration of instigated aggression against mating efficiency

#Model: Spearman rank correlation coefficient

#Result: rs = 0.074, N = 96, p = 0.477

#R Script:

srcc15 <- cor.test(aggmat$F.,aggmat$TDur,method='spearman')

srcc15

#Results:

#S = 136599, rho = 0.07352981, p-value = 0.4765

# 16) Lines 2350-233, 278-279

#Test: Average duration of instigated aggression against mating efficiency

#Model: Spearman rank correlation coefficient

#Result: rs = 0.080, N = 96, p = 0.438

#R Script:

srcc16 <- cor.test(aggmat$F.,aggmat$AvDur,method='spearman')

srcc16

#Results:

#S = 135630, rho = 0.08009709, p-value = 0.4379

# 17) Lines 233-235, 282

#Test: Experiment-level aggression (0-3) against mating success (1/0)

#Model: Binary logistic regression

#Result: z95 = -1.979, p = 0.048 *

# R Script:

blr17 <- glm(Mated~EC, data = aggmat, family = "binomial")

summary(blr17)

#Results:

#Estimate Std. Error z value Pr(>|z|)

#(Intercept) 2.3492 0.5044 4.658 3.2e-06 ***

#EC -0.5958 0.3010 -1.979 0.0478 *

# 18) Lines 233-235, 284

#Test: Experiment-level aggression (0-3) against mating efficiency

#Model: General linear model

#Result: t95 = 2.466, p = 0.016 *

# R Script:

glm18 <- glm(F.~EC, data = aggmat)

summary(glm18)

#Results:

#Estimate Std. Error t value Pr(>|t|)

#(Intercept) 1.22845 0.09836 12.489 <2e-16 ***

#EC -0.17241 0.06992 -2.466 0.0155 *

# 19) Lines 233-235, 284

#Test: Total experiences of aggression against mating efficiency

#Model: General linear model

#Result: t95 = -2.473, p = 0.015 *

# R Script:

glm19 <- glm(F.~X.E, data = aggmat)

summary(glm19)

#Results:

# Estimate Std. Error t value Pr(>|t|)

#(Intercept) 1.20099 0.08992 13.356 <2e-16 ***

#X.E -0.11164 0.04514 -2.473 0.0152 *

# 20) Lines 233-235, 287

#Test: Total experiences of aggression against mating success (1/0)

#Model: Binary logistic regression

#Result: z95 = -1.914, p = 0.056 .

#R Script:

blr20 <- glm(Mated~X.E, data = aggmat, family = "binomial")

summary(blr20)

#Results:

#Estimate Std. Error z value Pr(>|z|)

#(Intercept) 2.1763 0.4341 5.013 5.35e-07 ***

#X.E -0.3447 0.1801 -1.914 0.0556 .

# 21) Lines 233-235, 288

#Test: Total experiences of aggression against mating efficiency

#Model: Spearman rank correlation coefficient

#Result: rs = -0.227, N = 96, p = 0.026

#R Script:

srcc21 <- cor.test(aggmat$F.,aggmat$X.E,method='spearman')

srcc21

#Results:

#S = 180846, rho = -0.2265738, p-value = 0.02643

# 22) Lines 233-235, 289-290

#Test: Total duration of experienced aggression against mating success (1/0)

#Model: Binary logistic regression

#Result: z95 = -0.569, p = 0.569

#R Script:

blr22 <- glm(Mated~TDurE, data = aggmat, family = "binomial")

summary(blr22)

#Results:

# Estimate Std. Error z value Pr(>|z|)

#(Intercept) 1.745447 0.372464 4.686 2.78e-06 ***

#TDurE -0.007194 0.012634 -0.569 0.569

# 23) Lines 233-235, 289-290

#Test: Average duration of experienced aggression against mating success (1/0)

#Model: Binary logistic regression

#Result: z95 = -0.596, p = 0.551

#R Script:

blr23 <- glm(Mated~AvDurE, data = aggmat, family = "binomial")

summary(blr23)

#Results:

# Estimate Std. Error z value Pr(>|z|)

#(Intercept) 1.75155 0.37248 4.702 2.57e-06 ***

#AvDurE -0.02257 0.03785 -0.596 0.551

# 24) Lines 233-235, 291

#Test: Total duration of experienced aggression against mating efficiency

#Model: Spearman rank correlation coefficient

#Result: rs = -0.156, N = 96, p = 0.129

#R Script:

srcc24 <- cor.test(aggmat$F.,aggmat$TDurE,method='spearman')

srcc24

#Results:

#S = 170471, rho = -0.156207, p-value = 0.0.1286

# 25) Lines 233-235, 291-292

##Test: Average duration of experienced aggression against mating efficiency

#Model: Spearman rank correlation coefficient

#Result: rs = -0.162, N = 96, p = 0.114

#R Script:

srcc25 <- cor.test(aggmat$F.,aggmat$AvDurE,method='spearman')

srcc25

#Results:

#S = 171395, rho = -0.1624749, p-value = 0.1137

### Intersexual Aggression ##########

# (iii) Male-instigated intersexual aggression and mating success

# (Analyses 26-33)

# 26) Lines 239-243, 301

#Test: Behavioural consistency of instigating aggression against intersexual

# aggression (1/0)

#Model: Binary logistic regression

#Result: t95 = 0.597, p = 0.551

#R Script:

blr26 <- glm(Aggb~IC, data = aggmat, family = "binomial")

summary(blr26)

#Results:

# Estimate Std. Error z value Pr(>|z|)

#(Intercept) -1.9847 0.4784 -4.149 3.34e-05 ***

#IC 0.1909 0.3198 0.597 0.551

# 27) Lines 239-243, 301-302

#Test: Behavioural consistency of experiencing aggression against intersexual

# aggression (1/0)

#Model: Binary logistic regression

#Result: t95 = 1.832, p = 0.067

#R Script:

blr27 <- glm(Aggb~EC, data = aggmat, family = "binomial")

summary(blr27)

#Results:

# Estimate Std. Error t value Pr(>|t|)

#(Intercept) -2.4880 0.5327 -4.671 3e-06 ***

#EC 0.5770 0.3149 1.832 0.0669 .

# 28) Lines 239-243, 302-303

#Test: Total duration of aggression against intersexual aggression (1/0)

#Model: Binary logistic regression

#Result: t95 = -0.777, p = 0.437

#R Script:

blr28 <- glm(Aggb~TDur, data = aggmat, family = "binomial")

summary(blr28)

#Results:

#Estimate Std. Error t value Pr(>|t|)

#(Intercept) -1.56598 0.37155 -4.215 2.5e-05 ***

#TDur -0.01256 0.01615 -0.777 0.437

# 29) Lines 239-243, 302-303

#Test: Average duration of aggression against intersexual aggression (1/0)

#Model: Binary logistic regression

#Result: t95 = -1.018, p = 0.309

#R Script:

blr29 <- glm(Aggb~AvDur, data = aggmat, family = "binomial")

summary(blr29)

#Results:

# Estimate Std. Error t value Pr(>|t|)

#(Intercept) -1.46731 0.38953 -3.767 0.000165 ***

#AvDur -0.03728 0.03661 -1.018 0.308514

# 30) Lines 243-244, 305

#Test: Intersexual aggression (1/0) against mating success (1/0)

#Model: Chi-square

#Result: χ21 = 40.156, N = 96, p < 0.001 ***

# R Script:

table(aggmat$Mated, aggmat$Aggb)

cs30 <- chisq.test(aggmat$Mated, aggmat$Aggb)

cs30

#X-squared = 40.156, df = 1, p-value = 2.345e-10 ***

# 31) Lines 244-245, 305-306

#Test: Intersexual aggression (1/0) against mating efficiency

#Model: Wilcoxon rank-sum test

#Result: W = 979, p < 0.001 ***

# R Script:

wrst31 <- wilcox.test(aggmat$F.~aggmat$Aggb)

wrst31

#Results:

#W = 979, p-value = 9.253e-07 ***

# 32) Lines 245-247, 307

#Test: Intersexual aggression behavioural consistency (0-3) against mating

# success (1/0)

#Model: Binary logistic regression

#Result: z95 = -4.432, p < 0.001 ***

# R Script:

blr32 <- glm(Mated~Agg, data = aggmat, family = "binomial")

summary(blr32)

#Results:

# Estimate Std. Error z value Pr(>|z|)

#(Intercept) 2.7394 0.4613 5.938 2.89e-09 ***

#Agg -3.6091 0.8144 -4.432 9.35e-06 ***

# 33) Lines 245-247, 308

#Test: Intersexual aggression behavioural consistency (0-3) against mating

# efficiency

#Model: General linear model

#Result: t95 = -5.378, p < 0.001 ***

# R Script:

glm33 <- glm(F.~Agg, data = aggmat)

summary(glm33)

#Results:

#Estimate Std. Error t value Pr(>|t|)

#(Intercept) 1.15183 0.06021 19.129 < 2e-16 ***

#Agg -0.52880 0.09833 -5.378 5.49e-07 ***

### Figures ######################

## List graph colours

colours <- c("#FFFFFF", "#D3D3D3", "#747474", "#000000")

### Figure_1 ###

# Cricket line drawing figure (no associated code)

### Figure_2 ###

f2 <- subset(figs,select = c("TrialsE", "AvMatSucE", "AvMatSucESEProp"))

f2 <- na.omit(f2)

fig_2 <- ggplot(data = f2, aes(x = TrialsE, y = AvMatSucE))+

geom_col(col = "#000000", fill = "#FFFFFF")+

geom_errorbar(aes(ymin = AvMatSucE-AvMatSucESEProp,

ymax = AvMatSucE+AvMatSucESEProp), width = 0.2)+

theme_classic()+

ylab("Proportion of males that mated")+

xlab("Number of trials in which males experienced aggression")+

scale_y_continuous(limits = c(0,1))

### Figure 3 ###

# Figure_3a

f3a <- subset(figs, select = c("AggFBin", "AvMatSucFBin", "AvMatSucFBinSEProp"))

f3a <- na.omit(f3a)

f3a$AggFBin <- as.character(f3a$AggFBin)

fig_3a <- ggplot(f3a,aes(AggFBin,AvMatSucFBin))+

geom_col(col = "#000000", fill = "#FFFFFF", width = 0.5)+

geom_errorbar(aes

(ymin = AvMatSucFBin-AvMatSucFBinSEProp,

ymax = AvMatSucFBin+AvMatSucFBinSEProp), width = 0.1)+

theme_classic()+

scale_fill_manual(values = (colours))+

ylab("Proportion of males that mated")+

xlab("Male-instigated intersexual aggression")

# Figure_3b

f3b <- subset(figs, select = c("AggFBin", "MatEfFBin1Prop", "MatEfFBin2Prop",

"MatEfFBin3Prop", "NMFBinProp"))

f3b <- subset(figs, select = c("AggFBin", "MatEfFBin1Prop", "MatEfFBin2Prop",

"MatEfFBin3Prop", "NMFBinProp"))

f3b <- na.omit(f3b)

f3b$AggFBin <- as.character(f3b$AggFBin)

f3b <- rename(f3b, `High Efficiency` = MatEfFBin1Prop,

`Medium Efficiency` = MatEfFBin2Prop,

`Low Efficiency` = MatEfFBin3Prop,

`Never Mated` = NMFBinProp)

f3b_long <- gather(f3b, Number_Males,Frequency,`High Efficiency`:`Never Mated`)

f3b_long$Number_Males <- factor(f3b_long$Number_Males,

levels=c('High Efficiency',

'Medium Efficiency',

'Low Efficiency',

'Never Mated'))

fig_3b <- ggplot(f3b_long,aes(AggFBin,Frequency,fill = Number_Males))+

geom_col(col = "#000000", width = 0.5)+

theme_classic()+

scale_fill_manual(values = (colours))+

theme(legend.title = element_blank())+

theme(legend.position = c(0.8, 0.7))+

ylab("Proportion of males")+

xlab("Male-instigated intersexual aggression")+

scale_y_continuous(limits = c(0,1))

# Figure_3c

f3c <- subset(figs, select = c("AggF", "AvMatSucF", "AvMatSucFSEProp"))

f3c <- na.omit(f3c)

fig_3c <- ggplot(f3c,aes(AggF,AvMatSucF))+

geom_col(col = "#000000", fill = "#FFFFFF")+

geom_errorbar(aes(

ymin = AvMatSucF-AvMatSucFSEProp,

ymax = AvMatSucF+AvMatSucFSEProp), width = 0.2)+

theme_classic()+

scale_fill_manual(values = (colours))+

ylab("Proportion of males that mated")+

xlab("Instances of male-instigated intersexual aggression")

# Figure_3d

f3d <- subset(figs, select = c("AggF", "MatEfF1Prop", "MatEfF2Prop",

"MatEfF3Prop", "NMFProp"))

f3d <- na.omit(f3d)

f3d <- rename(f3d, `High Efficiency` = MatEfF1Prop,

`Medium Efficiency` = MatEfF2Prop,

`Low Efficiency` = MatEfF3Prop,

`Never Mated` = NMFProp)

f3d_long <- gather(f3d, Number_Males,Frequency,`High Efficiency`:`Never Mated`)

f3d_long$Number_Males <- factor(f3d_long$Number_Males, levels=c(

'High Efficiency', 'Medium Efficiency', 'Low Efficiency', 'Never Mated'))

fig_3d <- ggplot(f3d_long,aes(AggF,Frequency,fill = Number_Males), width = 0.2)+

geom_col(col = "#000000")+

theme_classic()+

scale_fill_manual(values=c("#FFFFFF", "#D3D3D3", "#747474", "#000000"))+

ylab("Proportion of males")+

xlab("Instances of male-instigated intersexual aggression")+

theme(legend.title = element_blank())+

theme(legend.position = c(0.8, 0.7))+

scale_y_continuous(limits = c(0,1))

# Figure 3 composite:

#1 x 2x2 vertical

#(Fig. 5: Panel A (TL): fig_3a, Panel B (BL): fig_3b,

#Panel C (TR): fig_3c, Panel D (BR): fig_3d)

# Key to above:

# TL = Top Left

# TR = Top Right

# BL = Bottom Left

# BR = Bottom Right

fig_3 <- grid.arrange(fig_3a,fig_3b, fig_3c, fig_3d, ncol = 2, nrow = 2)

### ALL FIGURES ###

fig_2

fig_3
