# Supplementary material for: Intrasexual aggression reduces mating success in field crickets
Source: Ecol Evol. 2023 Oct 1;13(10):e10557. doi: 10.1002/ece3.10557 (PMC10542478; doi:10.1002/ece3.10557)
Supplement: Supplementary file 2 — Table S1 [file ECE3-13-e10557-s002.docx]

**Table S1**

Expressions describing models used for statistical analyses in Main Text (top) and explanation of key terms (bottom).

| Eqn. | Full Model |
| --- | --- |
| 1 | *Aggression1 ~ i + Trial + (1\|Focal) + (1\|Interacting) + ε* |
| 2 | *I2 ~ i + I1 + (1\|ID) + ε* |
| 3 | *I3 ~ i + I1 * I2 * E1 * E2 + (1\|ID) + ε* |
| 4 | *E2 ~ i + I1 * I2 + (1\|ID) + ε* |
| 5 | *E3 ~ i + I1 * I2 * E1 * E2 + (1\|ID) + ε* |
| 6 | *Aggression1 ~ i + Trial Number + ε* |
| 7 | *Aggressive duration ~ i + #I + ε* |
| 8 | *Mating ~ i + Aggression2 + ε* |
| 9 | *Mating ~ i + Aggressive duration + ε* |
| 10 | *Mating ~ i + Experienced aggression + ε* |
| 11 | *Intersexual aggression ~ i + Aggression3 + ε* |
| 12 | *Mating ~ i + Intersexual aggression + ε* |

| Term | Explanation |
| --- | --- |
| *i* | *Intercept* |
| *Aggression1* | *Responses including the number and total duration of aggressive interactions* |
| *Aggression2* | *Number of aggressive instigations* |
| *Aggression3* | *Measures including the number of aggressive instigations, and their average and total duration* |
| *Aggressive duration* | *Measures including the average and total duration of all aggressive interactions* |
| *Ex* | *Experienced aggression in trial x* |
| *Experienced aggression* | *Measures including number of aggressive experiences, and their average and total duration* |
| *Intersexual aggression* | *Measures including overall expression of aggression (1/0)* |
| *Ix* | *Instigated aggression in trial x* |
| *Mating* | *Measures including mating status (1/0) and expediency* |
| *#I* | *Total number of aggressive instigations* |

**Table S2**

Results of GLMMs examining effects of prior instigated or experienced aggressive encounters on subsequent instigation or expression of aggression.

|  | **Response variable^1^** | | | | | | | |
| --- | --- | --- | --- | --- | --- | --- | --- | --- |
| **Factors^2^** | **I2** | | **I3** | | **E2** | | **E3** | |
|  | **z^3^** | ***p*-value^4^** | **z** | ***p*-value** | **z** | ***p*-value** | **z** | ***p*-value** |
| Intercept | -2.040 | 0.041 | -1.484 | 0.138 | -1.790 | 0.073 | -0.218 | 0.827 |
| I1 | 0.362 | 0.717 | 0.669 | 0.504 | -1.119 | 0.263 | 0.852 | 0.395 |
| I2 | - | - | 1.013 | 0.311 | - | - | -1.732 | 0.083 |
| E1 | 0.033 | 0.974 | 1.381 | 0.167 | 0.537 | 0.591 | -1.580 | 0.114 |
| E2 | - | - | -0.659 | 0.510 | - | - | -0.396 | 0.692 |
| I1*I2 | - | - | 0.158 | 0.874 | - | - | 0.097 | 0.923 |
| I1*E1 | 0.405 | 0.685 | -0.058 | 0.954 | 1.320 | 0.187 | 0.668 | 0.504 |
| I2*E1 | - | - | -1.610 | 0.107 | - | - | 1.517 | 0.129 |
| I1*E2 | - | - | 0.199 | 0.842 | - | - | -0.545 | 0.586 |
| I2*E2 | - | - | -0.167 | 0.867 | - | - | 0.026 | 0.979 |
| E1*E2 | - | - | -0.201 | 0.841 | - | - | 1.045 | 0.296 |
| I1*I2*E1 | - | - | 0.059 | 0.953 | - | - | -0.018 | 0.986 |
| I1*I2*E2 | - | - | -0.115 | 0.909 | - | - | <0.001 | 0.999 |
| I1*E1*E2 | - | - | 0.059 | 0.953 | - | - | 0.055 | 0.956 |
| I2*E1*E2 | - | - | 1.167 | 0.243 | - | - | -0.027 | 0.978 |
| I1*I2*E1*E2 | - | - | -0.066 | 0.947 | - | - | 0.011 | 0.991 |
| ^1^ Response variables were whether a focal individual instigated (I) or experienced (E) aggression in   trial 2 (I2, E2) or 3 (I3, E3).  ^2^ Factors describe whether the focal individual instigated (I) or experienced (E) aggression in   previous trials.  ^4^ Models are described in Eqns. 2-5 of Supplementary Table S1 (all n = 124). ^3^ Likelihood Ratio Tests using stepwise deletion were used to establish significance. | | | | | | | | |
